# Glucose Metabolism Reprogramming of Vascular Endothelial Cells and Its Implication in Development of Atherosclerosis

**DOI:** 10.31083/j.rcm2511423

**Published:** 2024-11-22

**Authors:** Shiwen Luo, Liu Li, Huiqing Chen, Jingyue Wei, Dongmei Yang

**Affiliations:** ^1^Key Laboratory of Vascular Biology and Translational Medicine, Medical School, Hunan University of Chinese Medicine, 410208 Changsha, Hunan, China; ^2^College of Integrated Chinese and Western Medicine, Hunan University of Chinese Medicine, 410208 Changsha, Hunan, China

**Keywords:** vascular endothelial cells, glucose metabolism, atherosclerosis

## Abstract

Atherosclerosis (AS) is an important cause of morbidity and mortality in cardiovascular diseases such as coronary atherosclerotic heart disease and stroke. As the primary natural barrier between blood and the vessel wall, damage to vascular endothelial cells (VECs) is one of the initiating factors for the development of AS. VECs primarily use aerobic glycolysis for energy supply, but several diseases can cause altered glucose metabolism in VECs. Glucose metabolism reprogramming of VECs is the core event of AS, which is closely related to the development of AS. In this review, we review how glucose metabolism reprogramming of VECs promotes the development of AS by inducing VEC barrier dysfunction, autophagy, altering the inflammatory response, and proliferation of VECs, in the hopes of providing new ideas and discovering new targets for the prevention and treatment of AS.

## 1. Introduction

Atherosclerosis (AS), is a chronic disease characterized by lipid deposition in large and medium-sized arteries and the eventual formation of plaques that block blood flow, and forms an important pathological basis for the occurrence of cardiovascular and cerebrovascular diseases [1]. This pathological process involves the abnormal proliferation and apoptosis of vascular endothelial cells (VECs), smooth muscle cells and fibroblasts, especially the early dysfunction of VECs [2, 3] and reprogramming of glucose metabolism, all of which are closely related to the occurrence of AS.

**Fig. 1.**
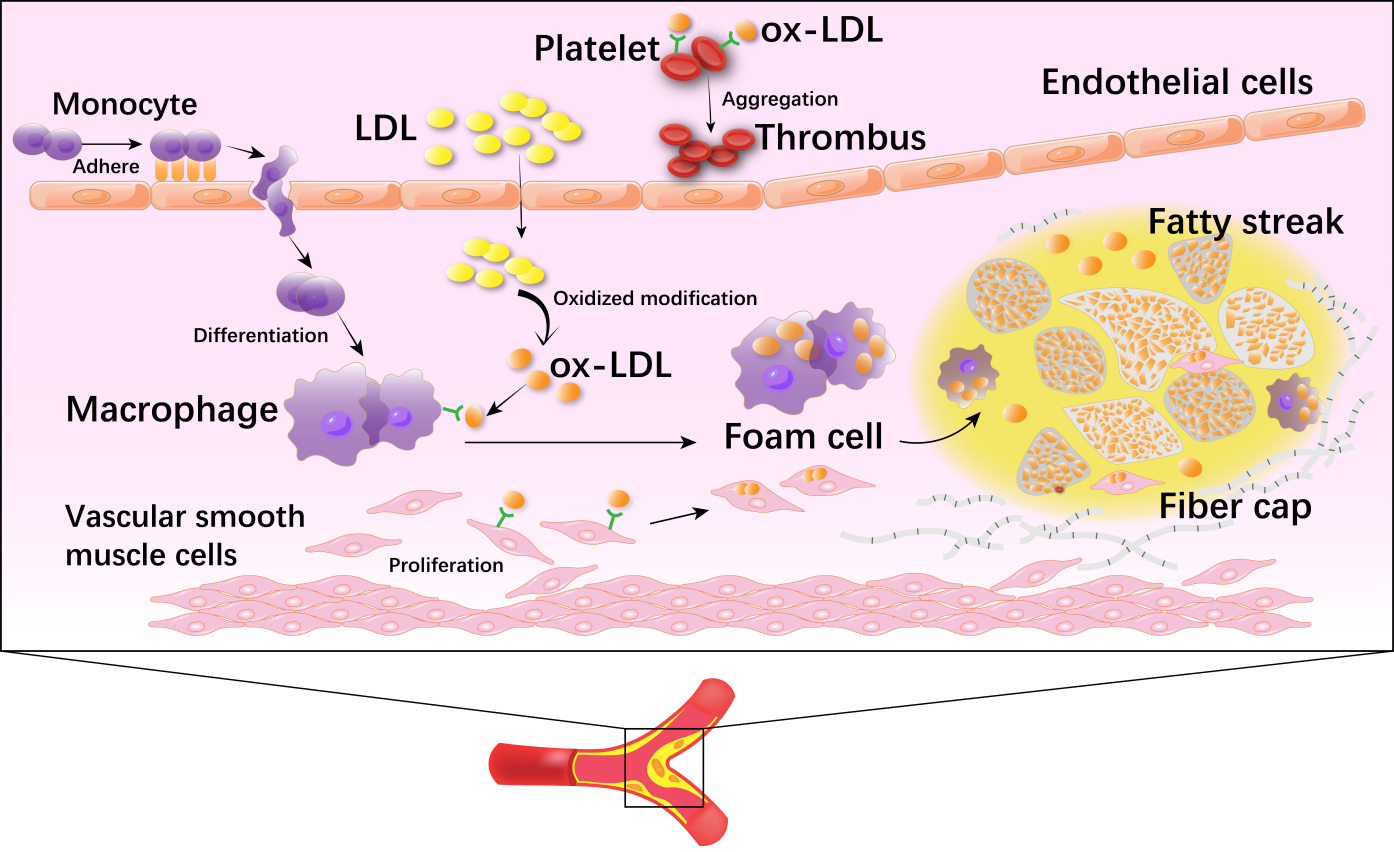
**The development of atherosclerosis**. Vascular endothelial cells (VECs) are damaged and destroyed. VECs recruit leukocytes and monocytes from the blood. Monocytes differentiate into macrophages after activation and engulf and digest oxidized lipoproteins to form foam cells. Foam cells gradually die and accumulate to form fatty streaks and plaques. Subsequently, fatty streaks and plaques, together with cholesterol crystals, dense collagen fibers, and scattered smooth muscle cells and other components, form a fibrous cap. LDL, low-density lipoprotein; ox-LDL, oxidized low-density lipoprotein.

In the early stages of AS, in the presence of risk factors such as hypertension, hyperglycemia, and nicotine from cigarette smoke, VECs are damaged and destroyed, leading to increased vascular endothelial permeability, which causes lipoproteins to aggregate in the subendothelial space. Simultaneously, VECs are activated to recruit leukocytes and monocytes from the blood [4]. Monocytes differentiate into macrophages after activation and engulf and digest oxidized lipoproteins to form foam cells. Foam cells gradually die and accumulate to form fatty streaks and plaques [5]. Subsequently, fatty streaks and plaques, together with cholesterol crystals, dense collagen fibers, and scattered smooth muscle cells and other components, form a fibrous cap, which eventually evolves into a plaque and promotes thickening and stiffening of the arterial blood vessel wall [6]. Therefore, the basic pathophysiological process of AS involves multiple cellular components (Fig. 1). Endothelial cell damage in the vascular wall is considered to be one of the risk factors for the occurrence of AS [7].

Thickness or hardening of the blood vessel wall and plaque formation caused by AS may obstruct blood flow, and leads to a variety of cardiovascular diseases. Currently, AS has become a major challenge to human health [8], and cellular dysfunction. Alterations in vascular development and structure caused by glucose metabolism reprogramming of VECs are closely related to the occurrence of AS [9, 10]. Therefore, the study of glucose metabolism reprogramming of VECs will provide new strategies for their prevention and treatment. This review summarizes the glucose metabolism reprogramming of VECs and its significance in the development of AS, providing new ideas for the prevention and treatment of AS.

## 2. VECs and Atherosclerosis

VECs are squamous epithelium located on the inner surface of blood vessels. As the primary natural barrier between blood and the vessel wall, VECs form a barrier through adherence and tight junctions between cells to avoid the entry of macromolecules such as lipoproteins and leukocytes. Adherence junctions are the more prevalent connection and are the determining factor for the barrier function of VECs [11]. Healthy VECs are usually stationary, but under the stimulation of hemodynamic changes, harmful substances and other factors, cell connections and their structural integrity are disrupted, which in turn provides the necessary conditions for lipid deposition, macrophage recruitment, and foam cell formation [12]. Therefore, damage to VECs is one of the initiating factors for the occurrence and development of atherosclerosis [13, 14, 15].

When quiescent VECs are activated, they accelerate the development of AS by causing platelet agglutination and stimulating platelets to secrete platelet-derived growth factors which promote abnormal proliferation of vascular smooth muscle cells to form fibromuscular plaques. Injured VECs release cytokines such as active monocyte chemotactic protein 1 (MCP-1), vascular cell adhesion molecule 1 (VCAM-1), P-selectin and E-selectin, to recruit monocytes to the subendothelium and induce monocytes to transform into pro-inflammatory macrophages. In turn, macrophages and the massive accumulation of foam cells exacerbate inflammation by releasing interleukin (IL)-1, IL-6, TNF-α, and Toll-like receptors to promote AS occurrence [16]. The inflammatory response also has an impact on thrombosis and plaque rupture. Inflammatory-responsive VECs release factors such as von willebrand factor (vWF), adenosine diphosphate (ADP), and collagen, which promote platelet aggregation and blood clot formation to accelerate the occurrence of AS [17]. In addition, activated VECs promote the proliferation of mesenchymal stem cells such as vascular smooth muscle cells and fibroblasts, and the latter induce the production of metalloproteinases and extracellular matrix proteins, which in turn promote plaque production [9].

Apoptosis of VECs has been found to be associated with the development of AS. Apoptosis of VECs causes a loss of their ability to regulate lipid homeostasis, immunity and inflammation. Oxidized low-density lipoprotein (ox-LDL) is considered to be one of the key factors in the induction of apoptosis in VECs. Ox-LDL produces lipid peroxides and induces apoptosis of VECs, inhibits phagocytosis of apoptotic cells and prevents the repair of VECs, promotes increased activity of the apoptosis-promoting proteins Bax, caspase9 and caspase3, and decreased expression of the anti-apoptotic protein b cell lymphoma 2 (Bcl-2) during oxidation [18]. The autophagic process is a self-protective mechanism to maintain the homeostasis of the intracellular environment. A study found that VECs which induce the formation of atheromatous plaques have the typical autophagic features such as increased expression of microtubule-associated proteins light chain 3 (LC3) and myelin-like structures, and increased vacuole formation [19]. Cav-1 deficiency plays a protective role by activating autophagic flux and attenuating the response of VECs to atherogenic cytokines [20]. In summary, damaged VECs and dysfunction of VECs including pro-coagulation, pro-inflammation, pro-proliferation, induction of apoptosis and autophagy, are closely related to the development of AS.

## 3. Glucose Metabolism of VECs

### 3.1 Normal Glucose Metabolism in VECs

**Fig. 2.**
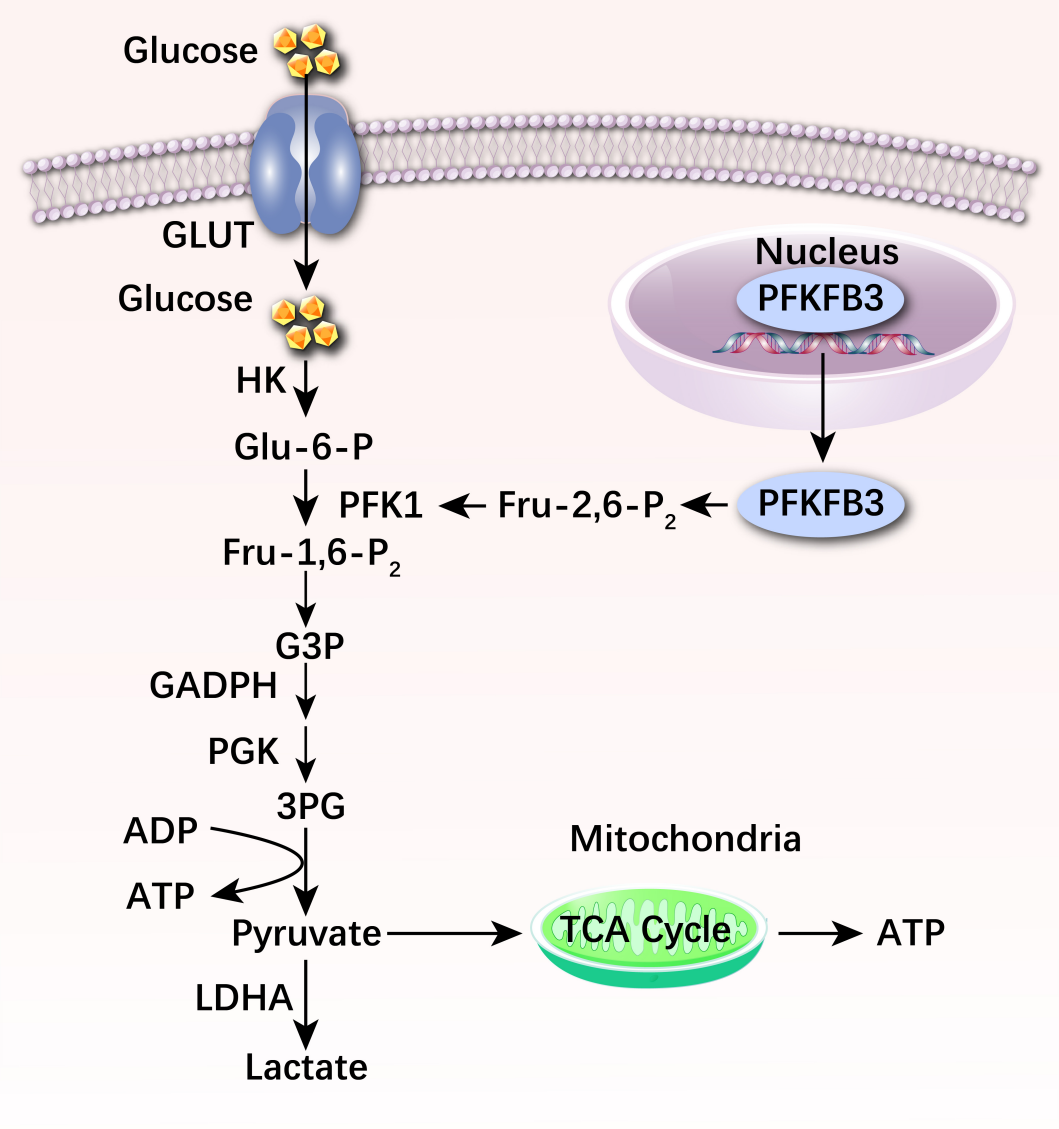
**Normal glucose metabolism process in vascular endothelial cells**. Absorbed into VECs, glucose catalyzes the production of glucose 6 phosphate (Glu-6-P) under the action of hexokinase (HK). Simultaneously, phosphofructokinase-2/fructose-2,6-bisphosphatase 3 (PFKFB3) generate 2,6-fructose diphosphate (Fru-2,6-P_2_), thereby activates the rate-limiting enzyme PFK1. Under the catalysis of PFK1, Glu-6-P phosphorylates to produce Fru-1,6-P_2_, and Fru-1,6-P_2_ is subsequently converted to pyruvate, which can be catalyzed to lactate by lactate dehydrogenase or enter tricarboxylic acid (TCA) cycle. GLUT, glucose transporter; ATP, adenosine triphosphate; GAPDH, glyceraldehyde-3-phosphate dehydrogenase; ADP, adenosine diphosphate; VECs, vascular endothelial cells; LDHA, lactate dehydrogenase A; PGK, phosphoglycerate kinase; G3P, glyceraldehyde 3-phosphate; 3PG, 3-phosphoglycerate; PFK1, phosphofructokinase 1.

Glucose is the main energy source of vascular endothelial cells. Glucose is first absorbed in the periphery of cells and aggregates at intercellular junctions. Subsequently, VECs will absorb glucose through a diffusion pathway mediated by glucose transporter 1 (GLUT1), which does not involve energy consumption [21]. After glucose is absorbed into VECs, it catalyzes the production of glucose 6 phosphate (Glu-6-P) under the action of hexokinase (HK). Simultaneously, glucose regulators phosphofructokinase-2/fructose-2,6-bisphosphatase 3 (PFKFB3), utilizing their high kinase activity to generate 2,6-fructose diphosphate (Fru-2,6-P2), thereby activating the rate-limiting enzyme phosphofructose kinase-1 (PFK1). Under the catalysis of PFK1, Glu-6-P phosphorylates to produce fructose 1,6-diphosphate (Fru-1,6-P2), and subsequently is converted to pyruvate, which can not only be catalyzed to lactate by lactate dehydrogenase, but also can be catalyzed to acetyl-coA delivery to the tricarboxylic acid (TCA) cycle [22]. The majority of normal cells in the body derive their energy from oxidative phosphorylation of the mitochondria. However, most tumor cells rely on aerobic glycolysis. Therefore, under aerobic conditions, they still choose the glycolysis pathway to generate energy, resulting in high glucose utilization and lactate secretion [23]. VECs also primarily use aerobic glycolysis for energy supply. Under physiological conditions, due to the inhibitory effect of glucose on mitochondrial respiration, less than 1% of glucose-derived pyruvate can be utilized in the TCA cycle, and this pathway accounts for only 15% of the total adenosine triphosphate (ATP) produced by VECs [24]. In comparison, up to 85% of ATP in VECs is produced by glycolysis, whose expression greater than glycolysis in other cell types [25]. Aerobic glycolysis results in far lower ATP production than oxidative phosphorylation. In addition, aerobic glycolysis can produce a large number of basic raw materials required for biosynthesis [3], therefore VECs prefer to rely on this energy metabolism pathway to meet their energy needs (Fig. 2).

### 3.2 Glucose Metabolism Reprogramming of VECs

Abnormalities of glucose metabolism of VECs is mainly characterized by increased expression and transcription of glycolytic genes, increased glycolytic flux, and significantly suppressed intracellular oxidative phosphorylation. A study found that mechanical low shear stress, hypoxia, hyperglycemia and several other specific environments can cause altered glucose metabolism in VECs [26].

VECs are simple squamous epithelium which are in direct contact with blood, and regulate their biological functions such as proliferation, differentiation and migration by sensing the mechanical forces generated by blood flow and translating them into corresponding biochemical signals [27]. Low shear stress (LSS) is the frictional force generated by blood flow impinging on the vessel wall, and its magnitude is closely related to the direction of blood flow, flow rate, and viscosity [17]. Arterial branches and curvatures where blood flow is uneven will induce LSS or oscillatory shear stress (OSS), leading to remodeling of the vessel wall, which results in VEC dysfunction, resulting in oxidative stress and endothelial metabolic dysfunction, resulting in phenotypic changes in VECs [28]. Meanwhile, low and disturbed shear stress (DSS) mediates the metabolism of VECs and promotes the AS process by reducing Krüppel-like transcription factor 2 (KLF2) through ox-LDL and hyperglycemia [29]. Compared with LSS, amp-activated protein kinase (AMPK) activated by pulsatile shear stress (PS) at straight parts of arteries can promote phosphorylation of the Ser-481 site of glucokinase regulatory protein (GCKR), resulting in decreased activity of hexokinase 1 (HK1), which attenuates glycolysis of VECs [30]. Study has found that Pim1 may effectively regulate the glycolysis of VECs through PFKFB3 in laminar shear stress [31]. In addition, the expression of hypoxia-inducible factor-1α (HIF-1α) was significantly upregulated after LSS in a murine carotid ligation model, and the expression of multiple glycolytic regulatory enzymes, including GLUT1 and PFKFB3, was upregulated. This implies that HIF-1α is required for the glycolytic enzymes in response to LSS [32].

Increased production of reactive oxygen species (ROS) and decreased nitric oxide (NO) in VECs are stimulated by hypoxic factors. NO can mediate the endothelium-dependent vasodilation required for normal vascular homeostasis and inhibit platelet aggregation, vascular smooth muscle cell proliferation and migration, and other key events in atherosclerosis [33]. Study has found that VECs cultured under hypoxic conditions had an increased rate of glucose transport and produced more lactate [34]. Interestingly, low levels of oxidative phosphorylation produce less ROS. Despite their proximity to a hyperoxic environment, VECs have reduced levels of oxidative stress, which protect it from ROS-induced cell death [35]. GLUT1 expression is upregulated in human umbilical vein endothelial cells (HUVECs) after being stimulated by hypoxia and promotes ROS-driven HIF-1α accumulation [36]. Furthermore, hypoxia leads to the expression of a series of glycolysis-related genes in VECs, such as the activation of pyruvate dehydrogenase kinase (PDK), which can inhibit pyruvate dehydrogenase (PDH), thereby mediating the conversion of pyruvate to acetyl-coA [37]. Lactate dehydrogenase A (LDHA) can promote the conversion of pyruvate to lactate, and the glycolytic flux of the pentose phosphate pathway in glucose metabolism is relatively increased. The activity of aerobic oxidation and the TCA cycle decreased, ultimately leading to the accumulation of pyruvate content in VECs, and the inhibition of the glucose oxidative phosphorylation pathway [38]. This transformation not only helps VECs fully absorb glucose, providing more energy for their proliferation, but also enhances metabolic-related cell signal transduction, thereby improving cell survival, similar to the Warburg effect of cancer cells. In addition, the upregulation of proline hydroxylase 3 (PHD3) was induced by hypoxia, and it binds to pyruvate kinase M2 (PKM2), which can also activate HIF-1α. Activated HIF-1α induces the transcription of important regulatory proteins in glycolysis, including GLUT1 [39], PFKFB3 [40], which increases the levels of glycolysis. The increasing glycolytic flux induces the proliferation of VECs and intraplaque angiogenesis. It may induce the release and aggregation of pro-inflammatory factors and increase atherosclerotic plaque instability, ultimately becoming potential factors for the development of AS.

Hyperglycemia is also one of the main causes of glucose metabolism reprogramming induced by VECs. It was shown that high glucose environment stimulated the increased expression of PFKFB3 protein in HUVECs. In contrast, inhibition of PFKFB3 significantly attenuated pathological angiogenesis induced by a high glucose environment and reversed the low expression of the protective factor phosphorylated protein kinase B (pAKT), in high glucose environments [41]. The expression of PKM2 was significantly upregulated in human retinal microvascular endothelial cells stimulated by high-glucose factors, and inhibition of PKM2 reversed the damage and apoptosis of VECs in high-glucose conditions [42]. In addition, hyperglycemia can induce cells to produce macromolecules and energy through glycolytic side pathways to maintain stable internal metabolic regulation [43]. The pentose phosphate pathway (PPP) and the polyol pathway (PP) are side branch pathways of glycolysis. Inhibition of glucose-6-phosphate entry into the pentose phosphate pathway under hyperglycemic conditions leads to reduced proliferation and migration of VECs. Glucose-6-phosphate dehydrogenase deficiency increases ROS by reducing glutathione (GSH), which in turn activates the transforming growth factor-β (TGFβ)/nicotinamide adenine dinucleotide phosphate (NADPH) oxidase/ROS signaling pathway to increase oxidative stress in human aortic endothelial cells [44]. Oxidative stress also exacerbates endothelial cell dysfunction, platelet activation, the coagulation cascade, and other pathways that promote thrombosis [45]. In addition, the polyol pathway is activated under hyperglycemic conditions, and the amount of glucose entering the pathway can increase up to 30%, while glucose is converted to sorbitol by aldose reductase, and sorbitol can be converted to 3-deoxyglucosone, a precursor of advanced glycosylation end products (AGEs) [46]. The overproduction of AGEs can result in numerous adverse effects in VECs by binding to the corresponding receptors, which affects the coagulation system, increases the permeability of VECs, inhibits endothelial nitric oxide synthase (eNOS) activity, and activates NADPH oxidase [47]. Hyperglycemia can also increase ROS levels by uncoupling eNOS and PPP, inhibiting glycolytic flux, and diverting intermediates to other metabolic pathways, leading to excessive production of ROS and AGEs [48].

**Fig. 3.**
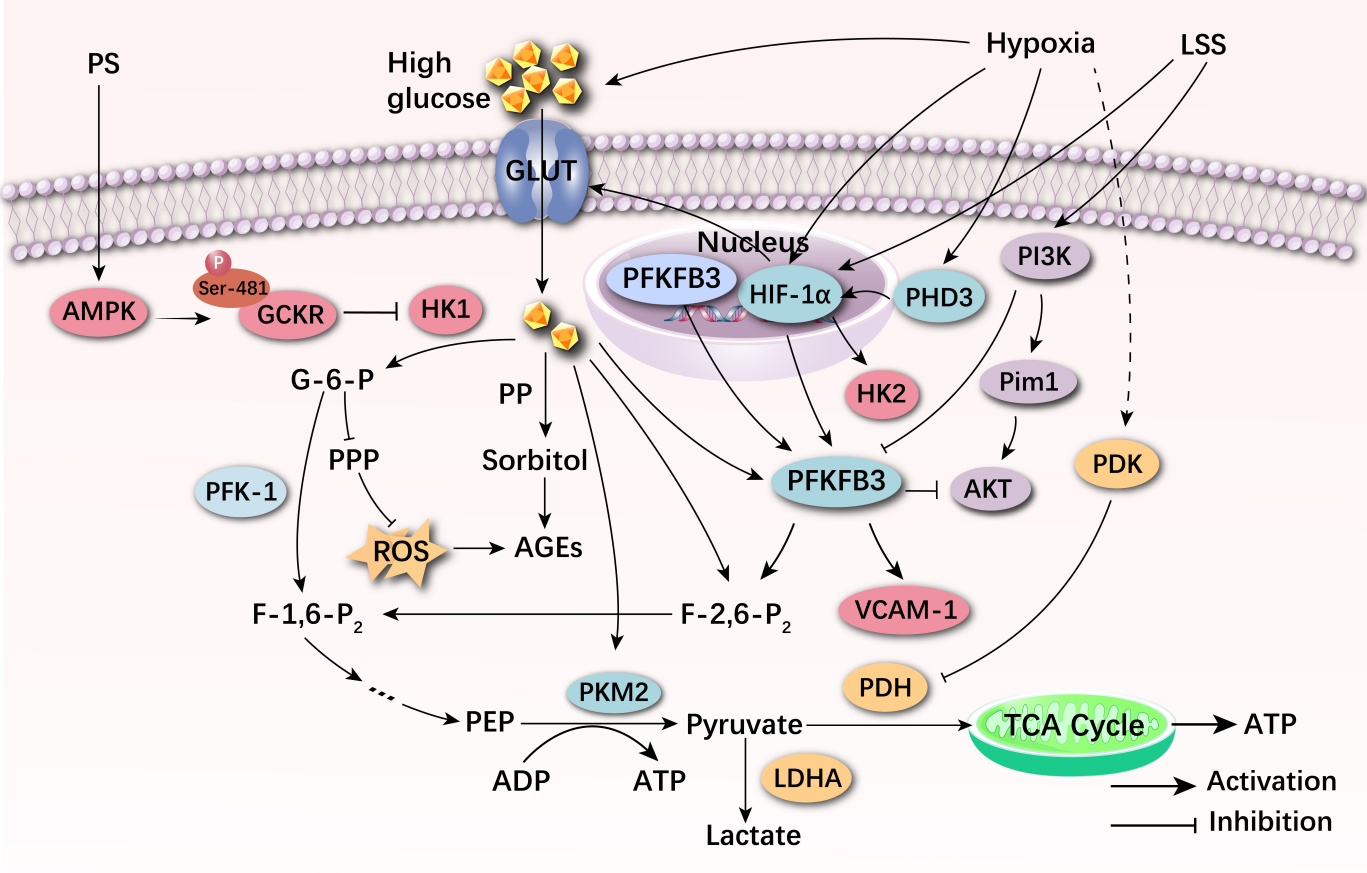
**Glucose metabolism reprogramming of VECs**. Pulsatile shear stress (PS) activated AMPK, which can promote phosphorylation of the Ser-481 site of GCKR to inhibit the activity of HK1. The expression of HIF-1α was significantly upregulated after imposing LSS. HIF-1α stimulates GLUT1 and PFKFB3. Stimulated by hypoxia, GLUT1 expression is upregulated and it promotes reactive oxygen species (ROS)-driven HIF-1α accumulation. Furthermore, hypoxia leads to the expression of pyruvate dehydrogenase kinase (PDK), which can inhibit pyruvate dehydrogenase (PDH). Lactate dehydrogenase A (LDHA) can promote the conversion of pyruvate to lactate. In addition, the activated proline hydroxylase 3 (PHD3) binds to pyruvate kinase M2 (PKM2), which can also activate HIF-1α. High-glucose factors stimulate the expression of PFKFB3 and PKM2. In addition, hyperglycemia can induce cells to produce macromolecules and energy through glycolytic side pathways. The pentose phosphate pathway (PPP) and the polyol pathway (PP) are side branch pathways of glycolysis. G-6-P is suppressed to entry into the PPP under hyperglycemic conditions, which leads to reduced proliferation and migration of VECs. Glucose-6-phosphate dehydrogenase deficiency increases ROS. In addition, the PP is also activated under hyperglycemic conditions, glucose is converted to sorbitol by aldose reductase, and sorbitol can be converted to 3-deoxyglucosone, a precursor of advanced glycosylation end products (AGEs). AMPK, amp-activated protein kinase; GCKR, glucokinase regulator; HK1, hexokinase 1; HIF-1α, hypoxia-inducible factor-1α; GLUT1, glucose transporter 1; LSS, low shear stress; PFKFB3, phosphofructokinase-2/fructose-2,6-bisphosphatase 3; PFK1, phosphofructokinase 1; PEP, phosphoenolpyruvate; ADP, adenosine diphosphate; ATP, adenosine triphosphate; TCA, tricarboxylic acid; AKT, protein kinase B; PI3K, phosphatidylinositol3 kinase; PHD3, prolyl hydroxylase 3; VCAM-1, vascular cell adhesion molecule 1; VECs, vascular endothelial cells.

VECs upregulate glycolytic fluxes to resist the hypoxic environment and maintain normal ATP levels under low shear stress, hypoxic stimulation, and stimulation by high glucose factors. In addition, glucose metabolism reprogramming of VECs may promote proliferation, oxidative stress, and inflammatory activation of VECs, which may ultimately lead to the development of AS (The process of glucose metabolism reprogramming of VECs is shown in Fig. 3).

## 4. Glucose Metabolism Reprogramming of VECs and Atherosclerosis

Glucose metabolism reprogramming of VECs is significantly related to the progress of AS. During the early stages of atherosclerosis, superoxide generated by NADPH oxidases originating from VECs plays a different role to the development of AS. NADPH oxidase 4 (NOX4) activates eNOS to improve vascular function, but accumulating ROS induces VEC barrier dysfunction and vasoconstriction to promote inflammation and thrombosis [49]. Under the stimulus of plaque composition such as ox-LDL, VECs and macrophages induce high glucose-induced NADPH oxidases to generate ROS, then upregulate the expression of adhesion molecules, which lead to altered glycolytic flux and accumulated intermediates. The generation of 2,3-bisphosphoglycerate is subsequently altered and influences the progress the atherosclerosis [50, 51]. Study suggests that vascular endothelial dysfunction takes place before the development of atherosclerosis. The first step in atherosclerotic plaque formation is that the inflammatory response is triggered and fatty streaks appear. Hyperglycemia induces the glycosylation of proteins and phospholipids, which subsequently increase oxidative stress. Products such as glucose-derived Schiff base and nonenzymatic reactive products form chemically reversible early glycosylation products, are rearranged to form more stable products, to produce AGEs after undergoing a series of complex chemical rearrangements. AGEs generate ROS which increase damage from oxidative stress and accelerate the process of atherosclerosis [52].

In susceptible areas of atherosclerotic plaques, the increased glycolytic flux of VECs can induce inflammation and other lesions, promoting further development of the middle stage of AS. Recently, it has been shown that blood flow turbulence can promote the glycolysis of VECs [53]. The disturbed flow in vulnerable regions prone to atherosclerosis enhances the expression and activities of protein kinase AMP-activated catalytic subunit alpha 1 (PRKAA1/AMPKα1), which support the expression of HIF-1α and the transcription of glycolytic enzymes, ultimately promoting the glycolysis of VECs. When cells are cultured with high glucose concentrations, more glucose is converted into pyruvate, resulting in the oxidation of electron donors such as Nicotinamide adenine dinucleotide (NADH) and flavin adenine adinucleotide reduced (FADH2) in the TCA cycle, inducing the excessive production of ROS and the development of atherosclerosis [25]. LSS activates HIF-1α through the non-classical pathway of nuclear factor κ-light-chain enhancer of activated b cells (NF-κB) and Cezanne to increase glycolytic enzyme expression in VECs. It then induces the proliferation of VECs and activates the inflammatory response leading to the development of AS [31]. The development of atherosclerosis leads to the stenosis of vessels resulting in the obstruction of blood flow which leads to hypoxia and ultimately tissue necrosis and organ failure. Simultaneously, VECs are able to perceive oxygen partial pressure and develop metabolic reprogramming by up-regulating glycolytic flux, in order to improve the rate of metabolism [25, 32].

The expression of HIF-1α, which plays a vital role during angiogenesis, is increased in advanced atherosclerotic plaques, and induces vascular endothelial growth factor (VEGF) and other growth factors, to enhance the perfusion of ischemic tissue to restore oxygenation [33]. HIF-1α-dependent induction of multiple glycolytic enzymes, which is involved in the process of ATP and macromolecules, are produced through the glycolytic pathway under anaerobic conditions. Hyperglycemia can induce dysfunction and apoptosis of endothelial cells, accelerating the development and rupture of atherosclerotic plaques.

## 5. The Mechanism of Glucose Metabolism Reprogramming of VECs in Atherosclerosis 

Several mechanisms of glucose metabolism in the reprogramming of VECs in atherosclerosis have been discovered, such as VECs barrier dysfunction, autophagy in VECs, inflammation of VECs, and proliferation of VECs. The detailed mechanism of glucose metabolism reprogramming of VECs in atherosclerosis are shown in Table 1 (Ref. [54, 55, 56, 57, 58, 59, 60, 61, 62, 63, 64, 65, 66, 67, 68, 69, 70, 71, 72, 73, 74, 75, 76, 77, 78, 79, 80]).

**Table 1.** **The mechanism of glucose metabolism reprogramming of VECs in atherosclerosis**.

Modeling method	Mechanism of action	Pharmacological action	References
Adeno-associated virus 1 × 10^12^ µg/mL	Pro-inflammatory signal pathway	Ox-LDL, LOX-1↓	[54]
		ICAM-1, VCAM-1↑	
		CCL5, CXCL1↑	
		ERK1/2, p38↑	
		Endothelial inflammatory reaction↑	
TNF-α 500 nM	NF-κB, PKC	SELE, VCAM-1, IL-6↓	[55]
		Intermediate factor↑	
		Endothelial inflammation↓	
DSS, LPS	S1PR2/RhoA/ROCK1	Endoplasmic reticulum stress↓	[56]
SiGLUT1	Lactic acid signaling pathway	MCT1, MCT5, MCT8↓	[57]
		Lactic acid metabolism↓	
BEG 10 µmol/L	PI3K/Akt/eNOS/NO antioxidant signaling pathway	ROS, LDH, MDA↓	[58]
		SOD↑	
		Akt ↓	
		eNOS phosphorylation↓	
		NO↓	
		Endothelial cell viability↓	
EGCG 100 µM	Glycolysis pathway	Angiopoietin-2 secretion↓	[59]
		Endothelial cell proliferation, migration, invasion↓	
		Barrier function↑	
Lactic acid 10 mM	HMGB1	YAP, SIRT1, HMGB1↓	[60]
		HMGB1 exosomes release↓	
Streptomycin 100 µg/mL	Glucose metabolism signal pathway	MCT1, MCT4↓	[61]
		VCAM-1, VE-cadherin↓	
		VEGFa, VEGFR2↓	
		TGF-β, IL-1β↓	
		Endothelial cell migration andproliferation↓	
		*In vitro* angiogenesis↓	
AGEs	Profilin-1 RhoA/ROCK1	RhoA, ROCK1↓	[62]
		ROS↓	
		Endothelial dysfunction↑	
		Oxidative stress↑	
FGF2 10 IU/mL	mTOR	P8 protein, ROS↑	[63]
		Mitochondrial membrane potential↓	
		Apoptosis↓	
		Angiogenesi↓	
Rapamycin	PI3K/Akt/mTOR	MiRNA-155, LC3-II↑	[64]
		P-mTOR/mTOR↓	
		Autophagy↑	
NAR 100 mg/kg	Inflammation signaling pathway	TC, TG, LDL-C, TNF-α↑	[65]
		ALT, MDA↑	
		HDL-C, SOD, GSH-Px↓	
		Autophagy↑	
ECGS 1%	MitoQ	NF-κB, NLRP3↓	[66]
		ROS↓	
		VE-cadherin decomposition↓	
		Actin cytoskeleton remodeling↓	
		Autophagy↓	
Ang-II	SESN2/AMPK/TSC2	mTOR phosphorylation↓	[67]
		Oxidative stress↓	
		Autophagy↑	
		Endothelial progenitor cell injury↓	
LDL 50 µg/mL	PI3K/Akt/mTOR	P62↑	[68]
		LC3-II, IR, LDLR↓	
		mTOR, Akt, GSK3β↑	
		Glucose uptake↑	
		Autophagy↓	
TNF-α 20 ng/mL	NF-κB	VCAM-1, ICAM-1↑	[69]
		LC3-II↑	
		Angiogenesis in plaque↓	
		Cardiac function↑	
Ang II	PKM2	EndoMT↓	[70]
		PKM2↑	
		Synthetic VSMC marker↓	
		Endothelial cell transformation↓	
Palmitate 200 µM	PINK1	P-Drp-1/Drp-1↓	[71]
		Fis1↓	
		Mfn2, Nix, LC3B↓	
		Oxidative stress↓	
		Mitochondrial autophagy↑	
Ox-LDL 50 µg/mL	Let-7e	lnc-MKI67IP-3↓	[72]
		IκBβ↓	
		NF-κB↑	
		Nuclear translocation↑	
		Inflammation↑	
		Adhesion molecule↑	
Amaryl novoNorm	Inflammation and oxidation pathway	GSH↑	[73]
		HsCRP, IL-6, TNF-α↓	
		MDA↓	
		Intima thickness of artery↓	
Glucan 40 mM	HIF-1α	ROS, NOX4, HIF-1α↑	[74]
		PDK1↑	
		Glycolytic enzyme↑	
		Inflammation↓	
		Mitochondrial respiratory ability↓	
E06-scFv 5 µg/mL	OxPL	TNF-α, IL-1β↓	[75]
		Cholesterol↓	
		Aortic valve calcification↓	
		Inflammation↓	
Toluene thiazine 20 mg/mL	PFKFB3	VLDL, LDL, HDL↓	[76]
		Glycolysis↓	
		GLUT3↓	
		Inflammation↓	
Cone plate flow system	PRKAA1/AMPKα1	PRKA, AMPK↑	[77]
		Glycolysis↓	
		Slc2a1, PRKAA1↑	
		Endothelial cell proliferation↓	
		Endothelial vitality↓	
EGF 0.1 ng/mL	TNF-α/NF-κB/HIF/VEGF	TNF-R1↓	[78]
		VEGF, HIF-1β↓	
		Angiogenesis↑	
		P65 phosphorylation↓	
Gibco 0.1 mg/mL	PFKFB3	MMP, dvWF↓	[79]
		VEGFR-2↓	
		Endothelial differentiation↑	
		Glycolysis↑	
		Angiogenesis↑	
Penicillin 100 U/mL	PFK15	Glucose intake↓	[80]
Streptomycin 100 µg/mL		Endothelial cell migration↓	
		Endothelial cell proliferation↓	
		Glycolytic activity↓	

Abbreviation: VECs, vascular endothelial cells; TNF-α, tumor necrosis factor-α; DSS, disturbed shear stress ; LPS, lipopolysaccharide; SiGLUT1, silencing glucose-facilitated diffusion transporter 1; BEG, β -elemene derivative; EGCG, espigallocatechin gallate; AGEs, advanced glycation end products; FGF2, fibroblast growth factor 2; NAR, naringenin; ECGS, endothelial cell growth supplement; Ang-II, angiotensin Ⅱ; LDL, low-density lipoprotein; Ox-LDL, oxidized low-density lipoprotein; E06-scFv, E06-single chain fragment of antioxidant phospholipid antibody; EGF, epidermal growth factor; NF-κB, nuclear factor κB; PKC, protein kinase c; S1PR2/RhoA/ROCK1, sphingosine phosphate receptor 2/Rho protein A/Rho-related kinase 1; PI3K/Akt/eNOS, phosphatidylinositol 3- kinase/protein kinase B/endothelial nitric oxide synthase; HMGB1, high mobility group protein1; mTOR, mammalian target of rapamycin; MitoQ, mitochondrial Q; SESN2/AMPK/TSC2, stress response protein 2/adenosine monophosphate activated protein kinase/tuberous sclerosis protein complex 2; PKM2, Pyruvate kinase M2 type ; PINK1, homologous phosphatase tensin induced kinase 1; Let-7e, Let-7 family regulator; HIF-1α, hypoxia inducible factor-1 α; OxPL, oxidized phospholipids; PFKFB3, fructose-2,6-bisphosphatase 3; PRKAA1/AMPKα1, protein kinase AMP-activated catalytic subunit alpha 1/5^′^-AMP-activated protein kinase catalytic subunit alpha-1; VEGF, vascular endothelial growth factor; PFK15, fructose -2,6- bisphosphatase 3 inhibitor-15; ICAM-1, intercellular adhesion molecule -1; VCAM-1, vascular cell adhesion molecule 1; CCL5, chemokine ligand 5; CXCL1, chemokine (C-X-C motif) ligand 1; ERK1/2, extracellular regulated protein kinase1/2; p38, P38 mitogen-activated protein kinase; SELE, selectin e; IL-6, interleukin 6; MCT1, monocarboxylic acid transporter 1; MCT5, monocarboxylic acid transporter 5; MCT8, monocarboxylic acid transporter 8; ROS, reactive oxygen species; LDH, lactate dehydrogenase; MDA, malondialdehyde; SOD, superoxide dismutase; YAP, yes-associated protein; SIRT1, silent information regulator 1; MCT4, monocarboxylic acid transporter 4; VEGFa, vascular endothelial growth factor a; VEGFR2, vascular endothelial growth factor R2 ; TGF-β, transforming growth factor β ; IL-1β, interleukin β; LC3-II, LC3-autophagy marker II; TC, total cholesterol; TG, triglyceride; LDL-C, low- density lipoprotein cholesterol; ALT, alanine aminotransferase; HDL-C, high density lipoprotein cholesterol; GSH-Px, glutathione peroxidase; IR, insulin resistance; LDLR, low-density lipoprotein receptor; P62, P62-chelate 1; EndoMT, endothelium-mesenchymal transforming factor; VSMC, vascular smooth muscle cells; P-Drp-1/Drp-1, p-dynamin-related protein 1/dynamin-related protein 1; Fis1, fission 1 mitochondrial protein; Mfn2, mitochondrial fusion protein 2; Nix, BNIP3-like Protein X; LC3B, light chain 3b; lnc-MKI67IP-3, antigen identified by monoclonal antibody Ki-67; IκBβ, inhibitor of nuclear factor κB inhibitory protein; GSH, glutathione; HsCRP, high sensitivity C- reactive protein; NOX4, nicotinamide adenine dinucleotide phosphate oxidase 4; PDK1, 3- phosphoinositide-dependent protein kinase 1; VLDL, very low-density lipoprotein; GLUT3, glucose transporter 3; Slc2a1, solute carrier family 2 member 1; PRKAA1, protein kinase AMP activates catalytic subunit α1; TNF-R1, tumor necrosis factor R1; HIF-1β, hypoxia inducible factor 1β; MMP, matrix metalloproteinases; dvWF, dvon Willebrand factor; VEGFR-2, vascular endothelial growth factor receptor 2; LOX-1, lectin-like oxidized low-density lipoprotein receptor 1; VE-cadherin, vascular endothelial cadherin; GSK3β, glycogen synthase kinase 3β; NLRP3, nlr family pyrin domain containing 3; HDL, high density lipoprotein; ↑, increase; ↓, decrease.

### 5.1 Glucose Metabolism Reprogramming Mediates VECs Barrier Dysfunction and Affects Atherosclerosis

Vascular endothelial barrier dysfunction is one of the factors in the development of AS. The main features of barrier dysfunction in VECs are disruption of intercellular junctions resulting in increased permeability, the entry of monocytes and leukocytes into the vessel wall causing structural damage, and the aggregation of plasma low-density lipoproteins under the endothelium [81]. In addition, a large number of neutrophils are recruited to VECs, resulting in an interaction between endothelial injury and the inflammatory response, which eventually leads to endothelial barrier dysfunction. There is convincing evidence that in the vascular endothelium, disintegrin and metalloproteinase 10 (ADAM10) can inhibit AS lesion occurrence by inhibiting pathological angiogenesis and ox-LDL-induced inflammation to regulate barrier function [54]. NF-κB and bromodomain-containing protein 4 can inhibit the expression of pro-inflammatory markers synergistically, such as VCAM-1 and IL6, and reverse the hyperpermeability induced by TNF-α [55]. The process of AS caused by vascular endothelial barrier damage can be mediated by multiple pathways including VEGF, tumor necrosis factor-1α (TNF-1α), AMPK, and interfering eNOS [82].

Glucose metabolism reprogramming plays an important role in mediating impaired vascular endothelial function. Study has found that activated pyruvate dehydrogenase complex (PDHC) reverses the glycolysis of HUVECs, while PDHC remains dephosphorylated to reduce lactate production and prevent HUVEC barrier dysfunction [56]. In turn, the glycolytic product lactate also has an effect on the barrier function of VECs. A study found that knocking down GLUT1 protein in mice, which decreases the accumulation of lactate in VECs, can lead to blood-brain barrier rupture and increased permeability [57]. Furthermore, in an endothelial barrier model constructed from HUVEC monolayers, the activation of σ1 enhanced endothelial barrier function and upregulated endothelial glycolysis [58].

In contrast, a study has also demonstrated that increased glycolytic flux promotes endothelial cell dysfunction and can damage barrier function by affecting the level of VE-calmodulin, particularly on endothelial cells [59]. Increased lactate induces extracellular signal-regulated kinase (ERK)-dependent activation of calpain1/2 for VE-calmucin hydrolysis, enhancing VE-calmucin endocytosis and increasing cell permeability in VECs [60]. 3PO (3-(3-pyridinyl)-1-(4-pyridinyl)-2-propen-1-one), as an inhibitor of glycolysis, can enhance the vascular barrier by inhibiting VE-calmucin endocytosis and promoting pericytes by upregulating N-calmucin adhesion, or inhibiting the expression of adhesion molecules in VECs by inducing NF-κB signaling pathway, and inhibit neovascularization and stabilize atherosclerotic plaques [61]. Hyaluronic acid nanoparticles (HA-NP) can penetrate into the plaque through the cellular junctions of VECs, but plaque HA-NP accumulation was reduced following treatment with 3PO, due to stabilization of barrier function. 3PO was found to significantly inhibit the activation and increase the continuity of VECs in *in-vitro* experiments [62]. In addition, inhibiting the sphingosine 1-phosphate receptor 2 (S1PR2)/Ras homologous genome members A(RhoA)/Rho protein-related curl spiral kinase-1(ROCK1) signaling pathway reduces altered barrier function in vascular endothelial cells via the downregulation of glycolysis [83].

In summary, glucose metabolism reprogramming, especially abnormal glycolysis, induces a bidirectional effect on the alteration of VECs barrier function. Glycolysis can not only reduce VECs barrier function damage, but also can promote VECs barrier dysfunction and increased permeability, which affects the development of AS, which is dominated by the protective effect on endothelial barrier function.

### 5.2 Glucose Metabolism Reprogramming Mediates Autophagy in VECs and Affects Atherosclerosis

Autophagy in VECs is a form of programmed cell death that participates in different stages of AS [84]. In the early development of atherosclerosis, autophagy in VECs stabilizes the formation of plaques and maintains endothelial integrity by reducing endothelial inflammation, oxidative stress, and endoplasmic reticulum stress [85]. In the late stages of atherosclerosis, due to insufficient autophagy, VECs are unable to clear the damaged organelles and denatured proteins, leading to increased intracellular oxidative stress and damage to VECs, promoting the development of AS [86]. Study has found that rapamycin can slow the formation of atherosclerotic plaques by activating autophagy [87]. Naringenin can inhibit inflammation and oxidative damage by inducing autophagy of VECs, thereby reducing the occurrence and development of AS [63]. These results indicate that normal autophagy levels are the main mechanism for delay of the development of AS. However, elevated levels of HUVEC autophagy induced by endostatin cause cell death [64]. Therefore, the autophagy of VECs has a dual role in regulating its survival and death. In addition, autophagy is also involved in the regulation of major functions in VECs, including angiogenesis, thrombosis, and NO production [65].

The relationship between autophagy and glucose metabolism in VECs has not been fully elucidated. A study found that the phosphatidylinositol3 kinase/protein kinase B (PI3K/Akt) signaling pathway not only induced GLUT1 transport into the cell membrane thus promoting glucose metabolism, but also inhibited autophagy induced by mechanistic target of rapamycin kinase (mTOR) and prevented the occurrence of cardiovascular diseases such as AS [66]. Endothelial cell (EC)-specific sirtuin 3 (SIRT3) transgenic mice reduced endothelial cell transition. Furthermore, knocking out SIRT3 could inhibit the maturation of the hyperacetylafion of endogenous autophagy-regulated gene5 (ATG5) and up-regulate the expression of PKM2 dimer, demonstrating that SIRT3 can regulate endothelial-mesenchymal transition by improving the autophagy degradation and glucose metabolism of PKM2 [67]. Study also indicated that the expression levels of Parkin, light chain 3 beta (LC3B)-II and beclin1 mitochondrial autophagy-related proteins were down-regulated in glucose/palmitic acid-treated rat aortic endothelial cells [68]. Additionally, the autophagy of HUVECs is significantly inhibited in a high glucose environment, manifested by up-regulation of Beclin-1, LC3-II protein and gene levels, and down-regulation of *p62* gene and protein expression levels [69]. Tumor protein 53 (TP53)-induced glycolysis and apoptosis regulator can effectively regulate the pentose phosphate pathway of VECs, up-regulate the expression of NADPH, and then inhibit excessive autophagy, which protects blood vessels [70]. Therefore, enhanced glucose metabolism can mediate the inhibition of autophagy and protect blood vessels. The inhibition of glycolysis can also treat AS by inhibiting autophagy of VECs. A study found that 3PO, a glycolysis inhibitor, could reduce the intracellular content of fructose 2,6-bisphosphate and inhibit the formation of coronary artery plaques in mice and improve cardiac function, via inhibiting the NF-κB/TNF-α signaling pathway and reducing the number of autophagosomes, which can down-regulate VCAM-1 and intercellular adhesion molecule-1 (ICAM-1). It has also been found that autophagy in VECs can affect glycolysis, i.e., inhibited autophagy increases GLUT1 expression in bovine aortic endothelial cells by approximately 50% [71]. Therefore, glucose metabolism reprogramming has a dual role in regulating the autophagy in the development of AS, in which the dominant effect appears to be that enhanced glucose metabolism inhibits the autophagy of VECs.

### 5.3 Glucose Metabolism Reprogramming Mediates Inflammation of VECs and Affects Atherosclerosis

In the early stage of atherosclerosis, the activated VECs stimulate various inflammatory cells, which secrete relevant inflammatory factors involving in all stages of AS [72]. The metabolic reprogramming of VECs plays an important role in this process, which contributes to increased expression of inflammatory genes. VECs exposed to disturbed flow can promote lactate production by upregulating glycolytic flux to activated macrophages and other immune cells via the NF-κB signaling pathway and ultimately promotes inflammation [88]. Similarly, study suggests that both inflammatory immune cells and VECs are subject to glycolytic remodeling, which interact with each other and are causally related [73].

Lipoproteins(a) [Lp(a)] can bind to plasma apolipoproteins(a) [Apo(a)] and form oxidized phospholipids (OxPLs), which plays a significant role in promoting inflammation in VECs [74]. Lp(a) has been shown to activate VECs by mediating PFKFB3 and GLUT1-regulated glycolysis [89]. The activated VECs further promote adhesion and migration of monocytes [75]. In addition, glycolysis mediated by PFKFB3 can help to activate macrophages and VECs, thereby causing inflammation and promoting the formation of atherosclerotic plaques [90]. Study also confirmed that treatment of VECs in mice with 3PO suppressed an inflammatory macrophage, M1, and promoted an anti-inflammatory macrophage, M2, to stabilize the atherosclerotic plaque [91]. The altered flow can activate HIF-1α regulated by NOX4-derived ROS, thereby promoting the expression of glycolysis-related enzymes and pyruvate dehydrogenase kinase-1 (PDK-1), and activating metabolic remodeling and inflammation of VECs [76]. Furthermore, VECs exposed to disturbed flows increased the expression of protein kinase AMP-activated (PRKA)/AMP-activated protein kinases (AMPKs), and activated HIF-1α to promoted the transcription of glycolytic enzymes and generation of macrophages [76]. Therefore, increased glycolysis in VECs can promote their inflammatory response. Conversely, glucose uptake and glycolysis will increase significantly when inflammatory factors stimulate VECs. Moreover, pro-inflammatory factors can activate and increase the expression of Yes-associated protein (YAP) and the transcriptional coactivator with PDZ-binding motif (TAZ). The pro-inflammatory YAP/TAZ signaling pathway can increase the level of glycolytic flux in VECs [77]. The production of TNF-α by VECs and macrophages is involved in activating the HIF pathway, resulting in enhanced levels of glycolysis in vascular endothelial cells, further confirming that inflammatory factors can also promote enhanced levels of glycolysis in VECs [92]. In conclusion, glucose metabolism reprogramming can promote the development of AS by mediating the inflammatory response in VECs.

### 5.4 Glucose Metabolism Reprogramming Mediates Proliferation of VECs and Affects Atherosclerosis

Under the influence of ischemia, hypoxia, inflammation and other factors, VECs can stimulate the release of VEGF and fibroblast growth factor (FGF). VECs change from a static state to an active state, allowing VECs to differentiate into terminal cells with high migration ability and stalk cells with proliferation ability, and then stabilize and lengthen new blood vessels. Additionally, glycolysis levels increase to provide energy for vascular development [78, 93, 94]. PFKFB3-mediated glycolysis plays an important role in endothelial differentiation and angiogenesis [95]. Alpha-mangosteen inhibits aerobic glycolysis, thereby reducing angiogenesis mediated by HIF-1α [79]. Study has shown that improving levels of glycolysis by blocking mitochondrial respiratory function can effectively promote tip cell differentiation and facilitate the formation of neovascularization [96]. In addition, PFKFB3 plays a key role in the glycolysis of VECs. PFKFB3-mediated glycolysis promotes VECs proliferation and pathologic angiogenesis in plaques [11, 97]. Pathological angiogenesis is the main characteristic of unstable atherosclerotic plaques, which is characterized by immature development and increased endothelial fragility. In addition, it may promote the exudation of endovascular red blood cells, which may lead to intra-plaque bleeding and greatly increase the risk of plaque rupture [98].

Ensuring that VECs have the ability to maintain the integrity of the vascular monolayer barrier has been found to be critical for the prevention of AS [99]. Researchers established an *in vitro* mouse model and found that PRKAA1 could enhance glycolytic flux and induce the proliferation of VECs by mediating the AMPK and the HIF-1α pathway, thus prevent the formation of vascular atherosclerotic plaques in mice [65]. In addition, collaboration between PFK15, a glycolysis inhibitor, and Sunitinib, a multikinase inhibitor, can inhibit the proliferation and migration of HUVECs [80]. During the process of neovascularization, the expression of VEGF is gradually reduced, and the expression of vascular inhibitory factor is increased to promote the formation of the basement membrane, so as to maintain vascular hyperplasia within the normal physiological range and promote plaque stability [100]. In addition, study has found that both Sirolimus and paclitaxel, stent drugs commonly used in the treatment of vascular restenosis, can inhibit the proliferation of hypoxic VECs by inhibiting glycolysis, thereby suppressing vascular inflammation and cellular mitochondrial dysfunction [101]. In conclusion, glucose metabolism reprogramming of VECs can stimulate their proliferation and migration, and play a dual role in the occurrence of AS, which can not only repair vascular intima, preventing further infiltration and accumulation of lipoproteins, but also induce neovascularization and reduce the instability of the AS plaque.

## 6. Potential Therapeutic Agents Targeting Endothelial Dysfunction

Evidence from basic and clinical studies suggests that clinically used drugs and drug candidates with varying structures and mechanisms of action can improve multiple aspects of endothelial dysfunction, and most of these drugs have shown promising cardiovascular protective effects in preclinical and clinical studies. Details of the studies (summarized) in this review, including drug names, mechanisms of action, clinical applications, and citations, are summarized in (Table 2, Ref. [102, 103, 104, 105, 106, 107, 108, 109, 110, 111, 112, 113]).

**Table 2.** **Potential therapeutic agents targeting endothelial dysfunction**.

Drug	Mechanism of action	Clinical application	References
Antihypertensive drugs (including ACEI, ARB, CCB, and β-blockers)	ARBs, CCBs, β blockers and ACEI:	Arterial blood flow disorders ↓	[102, 103, 104, 105]
	Endothelial function ↑	Ankle blood pressure ↓	
	ACEI and ARB:	The femoral vascular resistance ↓	
	ROS production ↓	Lipoprotein ↓	
	Vasoconstrictor production ↓		
	Endothelial function ↑		
VE-PTP inhibitors	Tie2 phosphorylation ↑	Endothelial barrier ↑	[106]
	eNOS ↑	Blood flow shear force ↓	
	VEGFR2 dephosphorylation	Blood pressure ↓	
Glycocalyx-targeted agents	NO-mediated vasodilation ↑	Arterial dysfunction ↓	[107]
	Endothelial function in mice ↑	Brachial artery flow-mediated dilation ↓	
eNOS enhancer (AVE3085), Antioxidants (including vitamin C/E, NAC, erdosteine, carbocysteine and genistein etc.)	The expression of eNOS ↑	Cardiovascular function ↑	[108, 109]
	NO production ↑	Vasomotor response ↑	
	Oxidative free radical ↓		
	Endothelial dysfunction ↓		
Anti-inflammatory drugs (including canakinumab, colchicine, inflammasome inhibitors, NSAIDs, resolvins)	Neutrophil chemotaxis ↓	Atherosclerotic thrombotic ↓	[110, 111]
	NLRP3 inflammasome ↓	CRP ↓	
	Endothelial inflammation ↓	LDL ↓	
		IL-1β↓	
Anti-diabetic drugs (including insulin, metformin, SGLT2i, GLP1RA, DPP-4i)	Pro-inflammatory mediators ↓	Blood glucose ↓	[112, 113]
	Vascular redox homeostasis is maintained	Frequency of hypoglycemia ↓	
	Endothelial function ↑	Glycosylated hemoglobin is stable in the normal range	
		Arterial dysfunction ↓	
		Blood pressure ↓	
		LDL ↓	
		Anti-platelet ↑	

Abbreviation: ACEI, angiotensin-converting enzyme inhibitor; ARB, angiotensin II receptor blocker; CCB, calcium channel blocker; VE-PTP, vascular endothelial tyrosine phosphatase; eNOS, endothelial nitric oxide synthases; NAC, acetylcysteine; NSAIDs, non-steroidal anti-inflammatory drugs; SGLT2i, sodium glucose cotransporter 2 inhibitor; GLP-1RA, glucagon-like peptide-1 receptor agonists; DPP-4i, DPP-4 inhibitors; ROS, reactive oxygen species; Tie2, angiopoietin-1 receptor tyrosine kinase; VEGFR2, vascular endothelial growth factor receptor 2; NO, nitric oxide; NLRP3, nlr family pyrin domain containing 3; CRP, c-reactive protein; LDL, low-density lipoprotein; IL-1β, interleukin-1 beta.

## 7. Conclusions and Perspectives

**Fig. 4.**
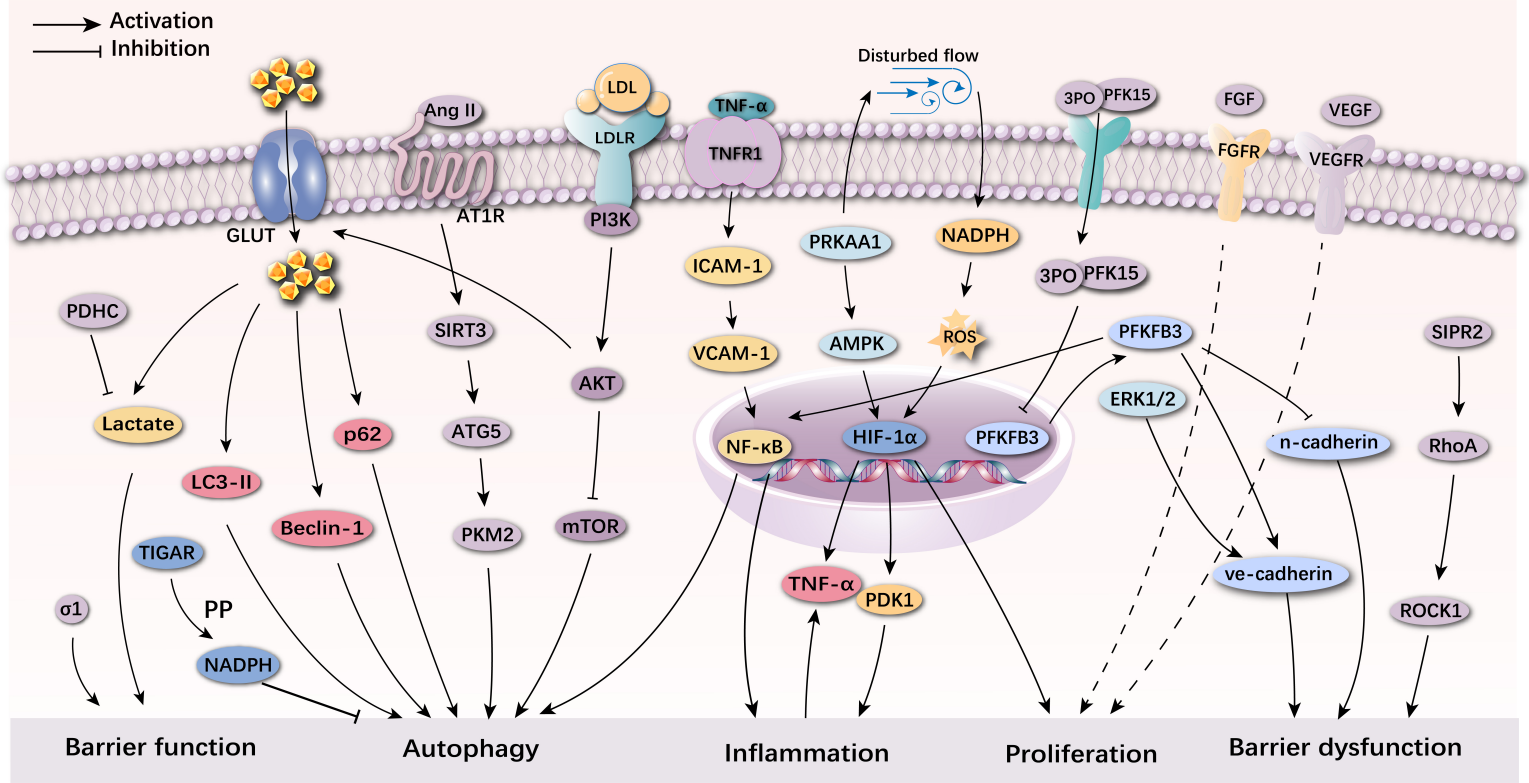
**Mechanism of glucose metabolism reprogramming of VECs in atherosclerosis**. Pyruvate dehydrogenase complex (PDHC) reduces lactate production and prevents barrier dysfunction. Furthermore, the activation of σ1 enhanced endothelial barrier function. In contrast, increased glycolytic flux promotes endothelial cell dysfunction and can damage barrier function by affecting the level of VE-calmodulin. Increased lactate induces ERK1/2 for VE-calmucin hydrolysis and increases cell permeability. PFKFB3 can break vascular barriers by upregulating VE-calmucin endocytosis, inhibit N-calmucin or upregulate the expression of NF-κB. The PI3K/Akt signaling pathway not only induces GLUT1, but also inhibits autophagy induced by mTOR. SIRT3 can stimulate ATG5 and up-regulate the expression of PKM2 dimers. Activated Beclin-1, LC3-II and inhibited p62 can stimulate the autophagy. Furthermore, PFKFB3 stimulates autophagy by activating NF-κB. In addition, glycolysis mediated by PFKFB3 can help the activation of macrophages and VECs, the disturbed flow can activate HIF-1α regulated by NOX4-derived ROS, thereby promoting the expression of glycolysis-related enzymes and PDK-1. Furthermore, PRKAA1 can enhance glycolytic flux and induce the proliferation of VECs by mediating AMPK and HIF-1α pathway. The production of TNF-α by VECs and macrophages is involved in activating the HIF-1α pathway. The proliferation of VECs can also be stimulated by activated VEGF and FGF. TIGAR, TP53 induced glycolysis and apoptosis regulator; LC3-II, microtubule-associated protein light chain 3 II; NADPH, transforming growth factor-β/nicotinamide adenine dinucleotide phosphate; p62, sequestosome 1; GLUT1, facilitative glucose transporter; Ang II, Angiotensin II; AT1R, angiotensin II type 1 receptor; SIRT3, sirtuin 3; ATG5, autophagy-related gene 5; PKM2, pyruvate kinase M2; AKT, protein kinase B; mTOR, mammalian target of rapamycin; PI3K, Phosphoinositide 3-kinase; ICAM-1, intercellular cell adhesion molecule-1; VCAM-1, vascular cell adhesion molecule 1; NF-κB, nuclear Factor kappa-light-chain-enhancer of activated b cells; PRKAA1/AMPK, 5’ AMP-activated protein kinase; HIF-1α, hypoxia-inducible factor-1α; TNF-α, tumor necrosis factor-α; PDK1, 3-phosphoinositide-dependent protein kinase-1; ROS, reactive oxygen species; PFKFB3, 6-phosphofructo-2-kinase/fructose-2, 6-biphosphatase 3; 3PO, 3-(3-pyridinyl)-1-(4-pyridinyl)-2-propen-1-one; PFK15, phosphofructokinase15; ERK1/2, extracellular regulated protein kinases; RhoA, ras homolog gene family, member A; ROCK1, rho-associated protein kinase1; SIPR2, sphingosine -1-phosphate receptor 2; LDL, low-density lipoprotein; LDLR, low-density lipoprotein receptor; TNFR1, tumor necrosis factor receptor 1; FGF, fibroblast growth factor; FGFR, fibroblast growth factor receptor; VEGF, vascular endothelial growth factor; VEGFR, vascular endothelial growth factor receptor.

Metabolism in VECs is of great interest but has been understudied. Glucose metabolism reprogramming of VECs can induce vascular dysfunction and the development of AS. Glucose is the main source of energy metabolism in VECs. Alterations in glucose metabolism under pathological conditions may also promote the development of AS. Glucose metabolism reprogramming of VECs in atherosclerosis promotes autophagy and inflammation which contributes to the formation and occurrence of AS. There are many pathways involved in glucose metabolism reprogramming of VECs (Fig. 4). Among them, glucose metabolism reprogramming of VECs mediated by the PI3K/Akt/mTOR pathway, the NF-κB/TNF-α pathway and the SIRT3 pathway are essential for suppressing autophagy in VECs. The glucose metabolism reprogramming of VECs mediated by the HIF-1α pathway and the PRKAA1/AMPK pathway have important roles in suppressing inflammatory responses in VECs. Glucose metabolism reprogramming of VECs mediated by NF-κB plays an important role in promoting VECs autophagy and inflammatory processes, while the PFKFB3 pathway plays an important role in both autophagy, inflammation and proliferation of VECs. The discovery of numerous targets related to glucose metabolism reprogramming of VECs is expected to provide new ideas for alleviating AS occurrence.
